# India-EU relations in health services: prospects and challenges

**DOI:** 10.1186/1744-8603-7-1

**Published:** 2011-02-10

**Authors:** Rupa Chanda

**Affiliations:** 1 Professor, Economics and Social Sciences Area, Indian Institute of Management Bangalore, India

## Abstract

**Background:**

India and the EU are currently negotiating a Trade and Investment Agreement which also covers services. This paper examines the opportunities for and constraints to India-EU relations in health services in the context of this agreement, focusing on the EU as a market for India's health services exports and collaboration. The paper provides an overview of key features of health services in the EU and India and their bearing on bilateral relations in this sector.

**Methods:**

Twenty six semi-structured, in-person, and telephonic interviews were conducted in 2007-2008 in four Indian cities. The respondents included management and practitioners in a variety of healthcare establishments, health sector representatives in Indian industry associations, health sector officials in the Indian government, and official representatives of selected EU countries and the European Commission based in New Delhi. Secondary sources were used to supplement and corroborate these findings.

**Results:**

The interviews revealed that India-EU relations in health services are currently very limited. However, several opportunity segments exist, namely: (i) Telemedicine; (ii) Clinical trials and research in India for EU-based pharmaceutical companies; (iii) Medical transcriptions and back office support; (iv) Medical value travel; and (v) Collaborative ventures in medical education, research, training, staff deployment, and product development. However, various factors constrain India's exports to the EU. These include data protection regulations; recognition requirements; insurance portability restrictions; discriminatory conditions; and cultural, social, and perception-related barriers. The interviews also revealed several constraints in the Indian health care sector, including disparity in domestic standards and training, absence of clear guidelines and procedures, and inadequate infrastructure.

**Conclusions:**

The paper concludes that although there are several promising areas for India-EU relations in health services, it will be difficult to realize these opportunities given the pre-dominance of public healthcare delivery in the EU and sensitivities associated with commercializing healthcare. Hence, a gradual approach based on pilot initiatives and selective collaboration would be advisable initially, which could be expanded once there is demonstrated evidence on outcomes. Overall, the paper makes a contribution to the social science and health literature by adding to the limited primary evidence base on globalization and health, especially from a developing-developed country and regional perspective.

## Background

Health services have become increasingly globalized. They are traded through all four modes of services delivery as defined under the General Agreement on Trade Services (GATS). Cross-border supply of healthcare takes the form of electronic delivery of healthcare across countries (GATS mode 1); consumption abroad takes the form of medical value travel (GATS mode 2); foreign commercial presence takes place through investments in the healthcare sector (GATS mode 3); and cross border movement of service providers involves the circulation of doctors and nurses among countries (GATS mode 4) The borderline between GATS modes may, however, not always be clear or separable (as in the case of electronic transactions involving modes 1 and 2) and all market segments may not be covered under the GATS. Globalization of health services has been facilitated by advancements in information and communication technology, liberalization of foreign investment, greater international mobility of patients and service providers, and demographic dynamics. As a result, today, health services are a subject of discussion in multilateral services negotiations.

The health sector has also come under focus in bilateral and regional trade and cooperation agreements. One such prospective accord is the India-European Union (EU) Trade and Investment Agreement (TIA) currently under negotiation. The latter is India's first agreement with a major developed country bloc and extends beyond goods into services, investment, and several other issues. This agreement could potentially facilitate India's growing bilateral trade and investment relations with the EU in services, including health services.

This paper examines the opportunities for and constraints to India-EU relations in health services. It identifies the various segments where there are opportunities for India to export health services to the EU and to collaborate with the EU. It also identifies numerous regulatory and other constraints which impede the development of this bilateral relationship. The discussion is largely based on in-depth discussions with a variety of stakeholders in India's health sector and official representatives from a few EU countries, corroborated by secondary evidence. In doing so, the paper makes a useful contribution to the social science and health literature by not only adding to the very limited information base available at present on globalization and health, based on primary evidence, but also by providing a North-South cum regional perspective.

The paper has two main conclusions. The first is that although India and the EU have very different health systems in terms of public-private composition, regulatory frameworks, and policy priorities, several factors make this sector conducive to expanding bilateral commercial relations and collaboration between the two. The EU member countries with their ageing populations, rising costs, and overburdened public healthcare systems could benefit from expanded relations with a country like India with its growing private healthcare sector, emergence of world class corporate hospitals, large pool of medical manpower, and young population across a variety of segments. The second conclusion is that given the nature of many of the constraints currently affecting this bilateral relationship and given the public good nature of health services, it would be best to take a gradual approach to expanding bilateral engagement in this sector, building on collaborative efforts selectively and over time moving towards more commercial engagements.

### Overview of the EU's Health Services Sector: implications for bilateral relations

Healthcare is a vital and strategic sector in the EU. On average, the EU spends close to 8 percent of its GDP on health [1]. Total healthcare spending in the EU-27 amounted to US $1.2 trillion in 2005, with France, Germany, and the UK constituting the three largest markets. The public sector dominates healthcare delivery. Public spending constituted 77 percent of total healthcare expenditures in 2005 for the EU-27 and close to 90 percent in certain EU countries [2]. The large volume of healthcare spending is indicative of this sector's strategic economic and social importance for the EU, which is likely to influence bilateral relations with other countries in this sector. The dominance of public spending in healthcare suggests that any bilateral discussions would be influenced by public sector concerns. In this regard, it is worth noting that although the EU has undertaken ambitious commitments on hospital services in its 1993/94 GATS schedule, it has reduced the coverage of these commitments under its Economic Partnership Agreement with the CARIFORUM to "privately funded services", reflecting the sensitivity surrounding commitment of publicly funded services in a trade agreement. Statements by the European Services Forum (ESF), which represents the interests of private sector services entities in the EU, similarly reflect the recognition of health and education services as special sectors where government plays an important role and that public health services must not be challenged by trade negotiations. According to the ESF, countries should be free to determine if they wish to open up their health services sectors to foreign providers.

The EU Member States provide universal or near-universal public coverage for health as part of a wider system of 'social protection'. This is extended to health services that are prescribed by health professionals or institutions registered with the health insurance system or which figure on the country's positive list of approved procedures, drugs, and medical devices. Private insurance offering 'supplementary' cover accounts for a small part of total healthcare financing, extending to services such as dental or alternative treatment which are not covered by the statutory systems, and providing supplementary coverage for elective treatments. The dominance of public insurance coverage has an important bearing on prospects for bilateral relations with non-member countries through modes such as medical value travel. It implies that issues of insurance coverage and portability are likely to be important and that the scope for medical value travel would be shaped by the efficiency and availability of health care under public health systems in the EU, and limited to areas where patients spend out-of-pocket or have limited insurance coverage.

Within the EU, nationals can elect to get treated in another member country for pre-approved procedures or in cases of undue delay, if they carry a European Health Insurance Card (EHIC), also called the EU Medical Card. The latter entitles its holders to receive treatment at reduced cost when visiting European Economic Area (EEA) countries and authorizes reimbursement by the patient's home country. It is important to note though that although the European Health Card facilitates treatment within the EU, it is subject to restrictions. It does not entail treatment on the same terms as those provided in the patient's home country and instead provides for treatment on the same terms as that provided to nationals of the host country. It also does not cover treatment for conditions existing before travel or treatment by private providers and few countries pay the full cost of treatment and travel insurance still remains necessary.

There is also an initiative to standardize health cards across member countries by providing an interoperable format that would help a patient prove entitlement to healthcare from different national health services or to medical insurance schemes in Member States. Such frameworks have implications for medical value travel prospects with non-EU countries versus EU member countries. Issues of level playing field between members and non-members are likely to feature in the EU's health services negotiations.

Another important aspect of the EU's healthcare system is the role of IT in healthcare delivery. The e-health industry in the EU was estimated at US $27.7 billion in 2006. Europe could potentially account for one-third of the global health ICT industry of US $66-79 billion [3]. Although the extent of IT integration in healthcare delivery varies across EU member countries, there is a general push in this direction due to ageing populations, rising operational costs, and the need to improve service access and quality. Several member countries have launched e-health initiatives. The adoption of IT in healthcare has implications for cross-border delivery of healthcare services to EU member countries, from within the region and outside, in areas such as teleradiology, telediagnostics, medical coding, transcriptions, and back-office support functions.

The EU market for e-health, however, remains fragmented with differences among member countries in their approach to IT adoption. There are also concerns about patient privacy, liability, and consumer safety, as reflected in very stringent data protection directives and regulations, at the EU and national levels [4]. The EU's Privacy Rule establishes regulations for the use and disclosure of Protected Health Information (PHI), which refers to any information about health status, provision of healthcare, or payment for healthcare that can be linked to an individual. The EU's General Directive on Data Protection is based on the principles of legitimacy, finality, transparency, proportionality, confidentiality and security, and control. This is supplemented by a Security Rule which deals specifically with Electronic Protected Health Information (EPHI) and specifies three types of security safeguards required for administrative, physical, and technical compliance, with security standards and specifications for each standard [5].

One important aspect of the EU's data protection directive pertains to data transfers to non-member countries [6,7]. It requires that Member States enact laws that prohibit transfer of personal data to countries outside the EU which fail to ensure adequate privacy protection. The Data Protection Commissions and Member States are required to inform each other in such cases. The data adequacy determination requirement and concerns over issues of data privacy and consumer protection have implications for cross-border electronic delivery of health services to the EU by non-member countries such as India and raise issues of level playing field vis-à-vis member countries.

Regulations concerning standards and eligibility requirements for healthcare providers in the EU are also likely to affect bilateral relations in health services with non member countries. There are requirements pertaining to registration, language certification, and insurance coverage, as well as compliance requirements with EU-wide as well as national-level legislation in areas such as telemedicine, clinical trials and research activities. Health professionals are regulated at the level of Member States and, to some extent, at the EU level [8]. There are two broad regimes for recognition of qualifications in the EU: (a) the sectoral system, based on common minimum training standards defined in relevant sectoral directives which lead to automatic recognition of the diploma; and (b) the "general system", which may require a case-by-case evaluation of the diploma by national authorities with the option to impose compensation measures [9]. Dentists, medical doctors, midwives, nurses, pharmacists and veterinarians are covered by the sectoral system; all other health professionals are covered by the general system [10]. These recognition requirements include competence assessment, certification requirements, specification of minimum training, and other conditions for the medical profession. Such regulations are likely to influence the ability of professionals and establishments to supply health services to the EU from outside the region as well as the portability of recognition within the EU given country-specific requirements [11].

Another important issue which is pertinent to the EU's bilateral relations with non-member countries is the exclusion of health services from the scope of the EU services directive. Notwithstanding initiatives to promote cross-border cooperation and to harmonize internal systems in healthcare among member countries, the latter retain their national legislation and regulatory frameworks to address concerns of consumer safety, standards, and accountability. To some extent the failure to have a single services market in the health sector reflects the wide variety in funding and delivery mechanisms that individual EU member states apply in their health care sectors and the extent to which competition between suppliers and insurers is admitted. This has implications for the extent to which the EU can be considered as a single bloc by third countries which wish to export to this region and also the extent to which the European Commission is in a position to negotiate on behalf of the entire Union. The non-applicability of the services directive to health care reflects a deeper problem of incompatibility among member states. Hence, negotiations in this sector are likely to be difficult and a selective engagement with a few markets within the EU may be more likely.

There are numerous challenges facing the EU's healthcare sector which have a bearing on its bilateral relations with countries like India. In a comprehensive report, the European Observatory on Health pointed out several issues facing the region's healthcare systems, including ageing populations and pressures on healthcare spending, limited human resources, the need to modernize and redesign national health services and improve management of the healthcare system, rising costs and unsustainable public health expenditures, long waiting times, and the need to give patients greater choice [12]. Such challenges potentially justify engagement through trade and collaborative arrangements within and outside the EU to alleviate these constraints. For example, long waiting times have resulted in increased pressure from patients in several EU countries to access services across borders. Sickness funds in some EU countries have contracted hospitals across borders to alleviate this pressure. In addition, there is demand for unauthorized and non-contracted care in other EU countries. In recent years, some EU countries have initiated reforms by undertaking quality assurance programs, providing guarantees of reduced local waiting times, facilitating intra-EU patient mobility and e-health, and initiating efforts to expand their health workforce, but the problems still persist. Hence, there are opportunities for providers in non-EU countries through outsourcing, medical value travel, movement of health personnel, and educational and research partnerships, which could potentially alleviate these cost and accessibility pressures. Regional agreements and collaboration could be used to facilitate such ties.

### Overview of India's health services sector: implications for bilateral relations

The Indian healthcare delivery market was estimated at US $34 billion and employed over four million people in 2008, making it one of the largest service sectors in the economy today. Total national healthcare spending stood at 4.1 percent of GDP in 2007 and is projected to double to 8 percent of GDP or $77 billion by 2012. The industry has grown at about 13 per cent annually in recent years and is expected to grow at 23 percent per year over the next few years [13]. Growth has been mainly driven by rising incomes, growing propensities to spend on healthcare, shift to lifestyle-related diseases, and demographic factors.

The sector comprises many segments. Estimates and projections for the individual segments show promising trends in several segments such as clinical trials, diagnostics, hospitals, medical devices, and health imaging. Nevertheless, India's healthcare sector falls well below international benchmarks for physical infrastructure, manpower, and existing standards in comparable developing countries. It is estimated that investment of $78 billion is required in health infrastructure and an additional 800,000 physicians are required over the next 10 years [14]. Considerable scaling up is required in the availability and quality of physical infrastructure and human resources.

One of the most important aspects of India's healthcare system is the significant role of the private sector, which accounts for over 75 percent of India's total healthcare spending. Private players account for 75 percent of dispensaries, 80 percent of all qualified doctors, and an estimated 95 percent of new hospital beds in recent years. Public health expenditure accounts for less than 1 per cent of GDP. Government spending on healthcare infrastructure (excluding land) is projected to rise only marginally, by 0.12 per cent of GDP and the private sector is expected to provide 88 per cent of investment requirements over the medium term [15]. However, private healthcare delivery is highly fragmented with over 90 per cent of it being serviced by the unorganized sector according to a recent report, and suffers from huge variation in quality and standards [16]. The growing dominance of private providers is significant for India's bilateral engagement with the EU. It suggests that the discussions are likely to be and are already being led on the Indian side by the private sector directly through industry consultations and delegation visits to these markets as well as being channelled through the government, while the counterparts in the EU are the governments and national health authorities. The latter in turn suggests potential conflicts of interests and concerns given the public-private nature of these discussions.

The regulatory environment in India's healthcare sector also has a bearing on its relations with other countries. Regulations in several areas pertinent to trade relations, such as standards for medical establishments, accreditation of medical professionals, and foreign direct investment are still evolving. Standards are currently being introduced for medical establishments, such as the recently introduced accreditation program for secondary and tertiary hospitals by the National Accreditation Board for Hospitals & Healthcare Providers (NABH) to improve the quality of healthcare establishments in the country, and which has also received international recognition by ISQua (International Society for Quality in Health Care). Similar standards have been prescribed for Indian laboratories by the National Accreditation Board for Laboratories to ensure compulsory registration of all clinical establishments and compliance with prescribed minimum standards, periodic inspections and inquiries, and cancellation of registration or penalties if conditions are not met. These recent efforts to establish regulatory frameworks and better governance mechanisms for healthcare providers are significant as they have a bearing on India's prospective discussions with other countries on issues of mutual recognition of standards and insurance portability.

Certification of medical professionals is another important issue that has a bearing on cross-border relations. Although India has established regulations at the central and state levels for medicine, dentistry, and nursing with rules for registration, practice, and enforcement of standards, there remain shortcomings. National level regulatory bodies and norms are lacking in areas such as paramedical services, standards and training tend to be non-uniform across educational establishments within the country, and there are no mutual recognition agreements with developed countries for qualifications of healthcare professionals. Such issues are likely to feature importantly in any efforts to develop bilateral relations with the EU in healthcare.

The globalization of India's healthcare sector in recent years has significance for India's cross-border engagements in health services, including with the EU. Rapid growth as well as the emergence of international quality private players in India's healthcare sector has created opportunities for trade, investment, and collaboration, cutting across all four GATS modes of delivery. According to secondary sources and discussions with industry experts, there are many existing and prospective opportunity segments for India to trade health services. With regard to mode 1, India has prospects in many aspects of e-health, including teleradiology, telediagnostics, telepathology, intensive care (or remote monitoring via tele-ICU), ophthalmology (remote diagnosis of eye problems), dermatology (remote diagnosis of skin problems), tele-psychiatry (using videoconferencing, TV cameras, and microphones to connect patients and psychiatrists for diagnosis, assessment, medication management and second opinions) and continuous online remote monitoring. These prospects are driven by India's cost advantage and the quality of its radiologists and specialized technical staff. Telehealth in these areas provides a means to address the shortage of physicians in the respective segments in the importing countries.

Independent telemedicine providers, reputed hospitals, and large Indian IT companies are currently providing telemedicine services to the US, Singapore, and several South and Central Asian countries. India is also an attractive market for healthcare business process outsourcing. Some reputed hospitals are partnering with US companies for billing, documentation of clinical and administrative records, coding of medical processes, and insurance claims processing services. Outsourcing of pathology services to India is another emerging opportunity area for Indian diagnostic labs.

India also has promising prospects in the area of medical value travel (mode 2). The medical value travel market in India was estimated at $333 million in 2004 and is projected to reach $2.2 billion by 2012 [17]. These prospects are driven by India's cost advantage, availability of world-class hospitals, and push factors in client markets. The cost of comparable treatment in India is on average one-eighth to one-fifth of those in the West and compares favourably with costs in other medical value travel destinations such as Thailand [18]. However, these exports remain constrained by lack of insurance portability and lack of accreditation of Indian healthcare providers by overseas health insurance trusts and private insurance companies.

Other segments where India is seeing growing opportunities are medical devices and clinical research and trials (in part facilitated by investments by overseas companies in India's health services and health products market). Many foreign companies are entering the Indian market through joint ventures and tie-ups in medical devices production and testing, training, and research. Some foreign companies conduct the first surgeries in India after the approval of a medical device or surgical treatment by their home authorities. The clinical research and trials segment has grown significantly with projected revenues of $1-2 billion by 2010 [19]. Some Indian research labs and Contract Research Organizations (CROs) provide sophisticated tests like molecular diagnostics for autoimmune disorders, cytogenetics and diseases related to abnormalities and also conduct bioequivalence studies. Some laboratories offer a wide menu of tests under one roof to foreign companies. Leading healthcare providers have received approval from overseas authorities to conduct clinical trials, including fast-track clinical trials.

India is also an established exporter of healthcare workers including doctors, nurses, and technicians (mode 4). Although much of this movement has been in the form of permanent migration, there are growing prospects for temporary movement of healthcare workers through institutional tie-ups with overseas establishments, to leverage India's cost advantage and manpower availability and also address the pressures of ageing populations and shortage of healthcare workers in developed countries. Non-uniformity of domestic standards of medical training, lack of mutual recognition, and immigration restrictions, however, constrain such prospects at present.

## Methods

There is little or no evidence on the current status of trade and investment flows between India and specific partner countries or regions such as the EU. The academic literature on bilateral relations in health care between India and specific countries is very limited, mostly consisting of industry and consulting firm reports with focus on specific segments.

This study relies on primary research, supplemented by secondary sources to understand the nature and extent of relations between India and the EU in health services. The primary survey consisted of 26 semi-structured interviews of a variety of stakeholders, including Indian health services firms, practitioners, government officials, and industry experts over the 2007-2008 period. The interviews were conducted in person and over the phone. The cities of Bangalore, Delhi, Kolkata, and Mumbai where major health service providers are located were covered.

The sample of healthcare establishments included leading Indian hospitals, telemedicine firms, clinical and specialized research firms, business process outsourcing firms in healthcare management, and medical equipment and technology firms. The practitioners covered include doctors, researchers, radiologists, biotechnologists, and senior management at health services firms. The segments and stakeholders were selected based on initial discussions with industry experts, other academics, and reading of secondary literature which helped identify both existing and prospective areas for India's trade in health services, not only with the EU but more generally. The interviews then specifically addressed the opportunities and challenges with respect to the EU. The aim of these discussions was to understand the range of services currently being provided by Indian providers to EU-based clients, the opportunities realized or perceived by them in the EU market, and the main barriers to doing business with the EU, including how the EU compared as a trading partner in this sector vies-a-vies other countries.

In order to validate these findings and to get alternate perspectives, views were also solicited from representatives in Indian industry associations, economic counsellors of the German and French embassies and the European Commission, and experts at the British High Commission based in New Delhi, and a UK-based medico-legal expert. Semi-structured and customized discussion guides were used for all interviews. The findings were presented at stakeholder consultations organized in New Delhi and Bangalore in February 2008 and 2009, respectively, and were strongly validated by participants. Further insights were also obtained at these consultations and incorporated.

Secondary research was used to gather background information on health services in India and the EU to understand key characteristics of this sector and their bearing on trade, investment, and collaboration opportunities between the two, as outlined in the preceding background section, and to corroborate the interview findings. Several health and economic databases (OECD and Eurostat) were also searched. Secondary information on India was primarily obtained from reports by industry associations, international agencies, researchers, consulting firms, and the popular media. The literature search focused on the post 2000 period.

## Results

This section provides an overview of the interview findings on the prospects and challenges concerning India-EU relations in health services and the general factors likely to shape this relationship.

### Overview of opportunities and constraints in the EU

The interviews indicated that bilateral commercial and other relations in this sector are very limited at present, also corroborated by the absence of data and studies in this regard. However, they also indicated several nascent and promising opportunity segments where bilateral engagement in the health sector could be developed. These were:

1. Telemedicine, most importantly teleradiology followed by telediagnostics, telpathology, bioinformatics, and continual remote monitoring;

2. Clinical trials and research in India for EU-based pharmaceutical companies and CROs;

3. Medical transcriptions, revenue cycle management, and other back-office support functions;

4. Medical value travel, especially for elective and out-of-pocket expenditures and alternative therapies and treatments;

5. Collaborative ventures between universities, hospitals, and research centres on medical education, research, training, staff deployment (especially nurses) and exchange, and product development under establishment-establishment arrangements and intergovernmental agreements

Broadly, two issues emerged regarding opportunities. First, respondents were generally more optimistic about expanding bilateral relations in non-intrusive areas and those with minimal patient contact and interface, i.e., the telemedicine, clinical trials and research, and back-office segments. Views were mixed regarding prospects in segments such as medical value travel or medical staffing as these were seen as directly subject to public perception and political, social, cultural factors that would be difficult to overcome in the EU.

Second, the discussion revealed that markets of interest to Indian healthcare providers vary within the EU depending on the opportunity segment in question. In telemedicine, the UK's National Health Service (NHS) was identified as the main client market for telemedicine exports from India while in the clinical trials and research segment, Germany and the Scandinavian countries were seen as important prospective markets due to their pharmaceutical base, inclination towards research and development, and acknowledgment of Indian expertise. In the area of personnel staffing and exchange, the UK (particularly the NHS) was identified as the main market, though potential was also perceived in the English language-inclined countries of Scandinavia, Germany, and the Netherlands. In medical value travel, apart from the UK, countries such as Germany, France, and the Scandinavian countries were seen as potential source markets given their inclination towards rehabilitative and alternative treatments and tourist interest in India. In general, the UK was seen as the main market for language and culture-dependent areas and emerged as the main market within the EU across almost all opportunity segments.

The interviews also revealed a variety of constraints faced by Indian healthcare providers in providing health services to the EU market. These pertained to regulation in EU Member States or at the EU-wide level, which included: (1) restrictions on outsourcing certain kinds of health services to providers outside the EU territory; (2) data protection and data exclusivity laws; (3) accreditation and certification requirements for healthcare establishments and compliance issues with international or EU standards and guidelines; (4) insurance portability restrictions and coverage issues; (5) recognition of professional qualifications and registration requirements; (6) immigration and visa regulations affecting mobility of providers; and (7) national treatment restrictions and discriminatory treatment which put Indian healthcare providers on an uneven playing field with EU-based providers and undermined their market access vis-à-vis competitor countries in the EU.

However, respondents made a distinction between *constraints *and *barriers*, clearly accepting that some regulations and requirements are warranted on public policy grounds such as protecting consumers, ensuring patient safety, and maintaining standards. In their view, it is often the associated administrative processes in the EU that create impediments as they are very cumbersome and time consuming, with approvals required from multiple institutions and regulatory authorities, and compliance requirements at the EU and country levels. The findings also highlighted the significance of social, linguistic, cultural, and perception-related factors in shaping the prospects for India-EU relations in health services, given the human resource-intensive and customer-service oriented nature of healthcare delivery. Both Indian and foreign respondents further highlighted regulatory, institutional, and infrastructural factors in India as constraining India's exports of health services to the EU market and the world market at large.

Broadly, two general factors emerged as key to shaping India-EU relations in health services. The first was awareness. Most Indian respondents noted that Indian healthcare providers have limited understanding about the healthcare sector in most EU countries excepting the UK's NHS. Since each EU country has its own complex and evolved healthcare system, according to the respondents, this lack of awareness within the Indian health provider community automatically constrains the scope for providing healthcare services to the EU market at large. Likewise, Indian respondents also pointed out that apart from the UK, there is limited awareness in the EU about the quality and capabilities of Indian health services providers.

The second factor that emerged as critical for shaping bilateral relations in health care was linguistic, social, and cultural affinity. Lack of such affinity between India and most EU countries was seen as a major constraint to India's delivery of healthcare and related services to the EU market. Respondents noted that healthcare is a highly personalized service where perceptions, attitudes, and social and linguistic ties play an important role. Thus, India's prospects were perceived to be limited to the UK market and a few EU countries that have English-speaking capabilities.

## Discussion

This section provides a detailed discussion of the survey findings for each of the identified opportunity segments. It highlights the existing status and prospects for bilateral engagement in each segment and associated constraints in the EU and in India.

### Prospects in Telemedicine

The interviews with Indian and EU respondents highlighted telemedicine, especially tele-radiology as one of the most promising areas for expanding bilateral relations, the key driver being the shortage of qualified persons and the launching of e-health initiatives in several EU countries. At present Indian firms do not provide telemedicine to the EU market as EU authorities do not deem India to be a data secure country. Hence, they do not permit outsourcing of patient data to India for telemedicine purposes. But discussions with management and practitioners at two leading Indian telemedicine establishments revealed that these data protection restrictions are expected to be removed eventually by the EU authorities once there is greater awareness of Indian providers and their capabilities and the cost advantages of outsourcing telemedicine become evident. This view was corroborated by secondary sources which indicated that several Trade Commissions from EU member countries have in recent years shown interest in outsourcing telemedicine work to India.

The interviews further revealed that some Indian firms are taking a long-term perspective and are adopting different strategies to circumvent these restrictions, for example, by establishing subsidiaries and partnerships within the EU in order to serve the EU market from within. Such commercial presence enables them to bid for teleradiology contracts that are being outsourced by some EU governments as their European subsidiaries are not subject to outsourcing restrictions on patient data. One leading Indian teleradiology firm confirmed that it has incorporated a subsidiary in the EU to undertake such work from within the EU and is also investing in a dedicated section at its India office to cater to prospective clients in the EU and gain a first-mover advantage in that market. Another leading Indian telemedicine provider has similarly used its overseas presence in the UK to tap the emerging business in telemedicine. It has a subcontract from a private consortium that has obtained a NHS contract for radiology reporting within the UK. The Indian firm has set up a local office in the UK staffed by Indian radiologists who are sent from India on a rotational basis to do the reporting work.

However, Indian providers noted four major constraints to providing telemedicine services for the EU, namely, data protection regulations, lack of recognition of the qualifications of Indian providers, contractual issues, and perceptions regarding India as a healthcare provider. The key aspects of these barriers and how they affect telemedicine exports from India to the EU are summarized in Table 1.

**Table 1:** Barriers affecting India's Telemedicine Exports to the EU

Constraint	Features and Implications
Data protection, privacy, and information security issues[21]	• Bureaucratic EU data protection laws • Cumbersome database registration requirement with data protection authorities • Data on EU patients cannot be sent outside the EU unless legal basis for transfer, i.e., official adequacy finding to determine country has national laws to provide adequate level of data protection • India has not received adequacy determination from EU authorities, so needs to legalize data transfer • Lack of harmonization in data protection legislation among members creates additional compliance costs of security audits, fines, registration in signing contracts with clients in different EU member countries • Stringent national level legislations on data and information security and data privacy relating to disclosure and use of Protected Health Information create additional administrative, physical, technical, and organizational compliance costs (e.g., need to adopt information security standards along the lines of the British Standard for Information Security management, BS-7799) • Firms may need to set up commercial presence in EU and provide telemedicine from within EU to overcome the absence of data adequacy determination for Indian providers based in India
Recognition and accreditation requirements	• Very expensive and time-consuming (as long as one year per provider) certification process • Multiple levels of verification with various professional bodies • Stringent certification requirements for teleradiology companies and providers • Registration required with each country's healthcare commission and concerned authorities • Compliance with EU directives on data protection, consumer safety, etc. • Indemnity/insurance requirement • Cumbersome evaluation and documentation requirements • Competence determination tests • Language requirements • Residency requirements • Requirement to appear in person for registration • Recertification, revalidation, re-licensure, regular appraisal requirements • Lack of harmonization within EU • Implicit discrimination against non-EU providers
Contractual issues	• Practical problems with malpractice insurance and liability policies in EU countries • Handling of breach of contract and jurisdictional issues in enforcing compliance • Costs imposed due to service line agreement clauses on prior consent, indemnity, non-disclosure, liability • Delays in executing contracts
Perception, attitudes, and stakeholder resistance	• Resistance to electronic delivery of healthcare in EU • Cultural and social barriers • Linguistic barriers, translation requirements for reports • Resistance from professional associations in EU due to concerns over employment losses

*Source*: Based on interviews

### Prospects in clinical research and trials

This segment, though nascent, was seen to be very promising for expanding commercial relations and collaboration between India and the EU. Some Indian companies are conducting clinical trials for European pharmaceutical companies. Some Indian CROs have set up marketing offices in a few EU countries, while others are acquiring companies in the EU and elsewhere to build their image and credibility. Although secondary data were not readily available to estimate the magnitude of this business with the EU, experts who were interviewed estimated that Indian companies were doing only some $100 million worth of clinical trials work for the EU compared to around $3 billion of work being done by the Eastern European countries.

However, as in the case of telemedicine, the interviews revealed that Indian companies are taking a long term view of the European market and plan to expand their business in the EU. Some Indian CROs are holding discussions with companies in the UK, Germany, and Italy. There has also been interest by Swedish, Danish, German, and Finnish companies about conducting clinical research and trials in India for faster turnaround. Some areas of interest for European companies are Phase I and II studies on diabetes, oncology, neuropsychiatry, gastroenterology, and stem cell research. Respondents also noted that there are ongoing discussions with European biopharma companies for proof of concept for new drugs. European countries with the strongest pharmaceutical sectors, namely the UK and Germany, were seen as the most important markets in the EU. Indian firms also noted the scope for research in experimental therapies that could be conducted by Indian companies or research centres in collaboration with European institutions and universities and for potential partnerships between Indian and EU laboratories to get international certification for evaluation and testing.

According to respondents, the driving force for expanding India-EU relations in the clinical trials and research segment would be the high drug development costs, the limited patient pool, and slow recruitment rate of patients for clinical trials in the EU. It was noted that India is cost-effective for conducting clinical trials given its huge population, diverse genetic pool, wide range of diseases, drug-naïve population, trained medical and technical manpower, and good hospitals for undertaking such trials. According to these respondents, Indian CROs can help EU-based pharmaceutical companies lower their costs and the time to market drugs.

Several constraints were also highlighted, though these were often seen as necessary regulations and not barriers per se. These constraints mostly pertained to data exclusivity requirements, accreditation and certification requirements for laboratories and organizations conducting the trials, and contractual obligations. The interviews also revealed perception-related barriers due to the lack of awareness in the EU about India's capability as a destination for clinical trials and research. There were also concerns expressed by EU officials on ethical grounds. Table 2 summarizes the main constraints that emerged with regard to clinical trials and research, and their resulting implications for Indian companies.

**Table 2:** Constraints affecting clinical trials and research

Problem	Features and Implications
Standards and Accreditation	• Requirement to conform with client country guidelines often cumbersome • Accreditation of Indian labs required even if they conform to accepted global standards • Compliance costs of meeting documentation, audit, infrastructure, qualification, training requirements
Norms for clinical trials	• Stringent requirements for informed consent, transparency, adherence to prescribed norms
Data Protection	• India not perceived as data-secure • Data exclusivity contracts have to be signed • Detailed audits required • Costs of litigation
Manpower mobility	• Problems in getting visas for technical persons sent by Indian CROs to clients in EU-- short duration, single entry

*Source*: Based on interviews

### Prospects in Medical Value Travel

To date, India's medical value travel exports are mostly to developing countries in South Asia, Africa, and the Middle East. Interviews with practitioners and management at leading Indian hospitals indicated that there are very few medical value travellers from the EU to India. The latter are limited to out-of-pocket patients and elective treatments. However, respondents were optimistic about the prospects for expanding medical value travel from several EU countries, especially the UK, given the latter's colonial, linguistic, and social ties with India. This view was corroborated by secondary sources where according to a survey conducted by the Treatment Abroad website in 2007, over 70,000 British citizens who travelled abroad for medical treatment noted India as a destination of choice [20].

Some Indian practitioners also cited prospects in certain countries of Eastern Europe, such as Poland, which face challenges in their healthcare system following their transition from socialism. They pointed to possibilities in the form of commercial presence by Indian hospitals or tie-ups with institutions in these countries, given the latter's need for affordable healthcare, lack of quality medical infrastructure, exodus of medical personnel to Western Europe following accession, and possible affinity to India due to good political relations in the past.

However, the general view was that developing and least developed countries rather than developed regions such as the EU would continue to be the main sources for medical value travellers to India. There was also generally much greater optimism among all respondents about the prospects in alternative medicine and therapies given growing interest in the West for treatment of chronic disorders where allopathy fails to deliver. Respondents noted that India has the potential to provide various streams of alternative medicine, including panchkarma, ayurveda, unani, siddha, and homeopathy. This finding was corroborated by rough estimates provided by some respondents on the share of European patients seeking treatment at traditional allopathic versus alternative treatment facilities. The share of European patients at alternative treatment facilities was over 50 percent in some cases while in all the traditional corporate hospitals that were covered by this survey, this share was less than 10 percent.

The in-depth discussions also pointed out various factors which limit and will probably continue to limit medical value travel from the EU to India. These related to:

(i) Restrictions on reimbursement of patients from the EU if travel to the exporting country exceeds a certain duration, effectively affecting India's attractiveness as a medical destination;

(ii) The relatively low share of non-insured and out-of-pocket paying patients in the EU that automatically limits the pool of patients who would opt for treatment in India;

(iii) Dominance of the public sector as a provider of insurance which creates problems of political acceptability in allowing medical value travel to India and getting reimbursed by the national health insurance trusts in EU countries;

(iv) Lack of accreditation of Indian hospitals and the lack of recognition of Indian medical qualifications which affect the scope for reimbursement for treatment in India.

In addition, respondents noted the role of linguistic, cultural, and social differences in limiting India's medical value travel exports to the EU. They also stressed the importance of perception given the fact that medical value travel involves a close interface between the doctor and the patient. In their view, attitudinal factors and India's lack of credibility as a medical value travel destination is likely to remain a constraint to such exports to the EU. Table 3 summarizes the main constraints to expanding medical value travel from the EU to India.

**Table 3:** Constraints to India's Medical Value Travel Exports to the EU

Problem	Features and Implications
Insurance portability regulations	• State insurance trusts and private insurance companies do not accept treatment in India for reimbursement • Flight time restrictions for UK patients (limited to 3 hours) for reimbursement from NHS • Restrictions on reimbursement of alternative medicines and therapies for lack of scientific evidence and registration
Growing competition	• India at disadvantage relative to Eastern European countries on qualification, e-health delivery, movement of persons, insurance portability
Perceptions	• Nationally sensitive issue, resistance to medical value travel by national health providers • Cultural, social, linguistic perceptions about India • Perceptions about India as a suitable destination for medical value travel

*Source*: Based on interviews

### Prospects for back-office support services

One interesting opportunity segment that emerged from the in-depth discussions was back-office business process and support services in healthcare delivery and administration. The interviews highlighted the existence of such exports by Indian firms for the US market and similar prospects for doing high-end, back-office work in healthcare for the EU market.

One specific activity that was cited was revenue cycle management, which involves taking patient bills and records for processing reimbursements from insurance companies. Respondents noted that such services involve specialized expertise and that Germany has recently expressed an interest in outsourcing medical transcription as well as other IT-enabled services to India to overcome its high costs and labour shortages in healthcare. Another activity where Indian firms could provide specialized business process support services was medical coding and analysis of patient charts to ease reimbursement-related analysis by insurance companies. The interviews highlighted the prospects for providing such coding services to the EU for data analysis and diagnostic purposes, based on the European Procedural Terminology. However, the discussions also highlighted several constraints to India's exports of back office health support services to the EU, several of them common to other opportunity segments. These constraints and their implications are summarized in Table 4.

**Table 4:** Constrains to India's provision of support services in healthcare to the EU

Problem	Features and Implications
Accreditation	• Certification required by concerned regulatory bodies in various segments (medical coding, analysis) • Additional requirements of continuing certification and evaluation
Data privacy and restrictions on international data transfer	• India is not empanelled as a data-secure by EU authorities • Restricts scope for data transfer and related outsourcing • Compliance costs of meeting EU and individual countries' data protection legislation
Limited scope of the EU	• Resistance to outsourcing of back-office functions in the EU

*Source*: Based on interviews

### Prospects for collaboration in training, research, and staffing

The interviews also indicated several areas for India-EU collaboration in health services. Given the shortage of personnel in several EU countries, respondents noted that India could export medical personnel on a temporary basis to staff the national health systems of those countries, particularly for nursing and paramedical services. The UK was cited as the most important prospective market for deployment of health personnel.

Another potential area for collaboration, highlighted by the interviews was medical education and training. While the EU countries have thus far shown little interest in entering India's medical education segment, both Indian and EU respondents noted that there are possibilities for collaboration through technical tie-ups, dual degrees, and twinning programs, which could be combined with a period of deployment and practical training in the EU following coursework. Indian respondents also highlighted the fact that since the EU has excellent hospitals with trained personnel and established processes in subspecialty care, collaboration in post-graduate training would help raise Indian standards while also addressing labour shortages in those countries. Danish authorities have expressed interest in such collaboration. EU companies engaged in the development and production of medical equipment and devices could potentially be part of this collaboration by partnering with academic institutions and healthcare providers in India for research and development and training services.

The interviews also indicated possibilities for collaboration in knowledge process outsourcing of specialized and technical services for the healthcare industry, such as design and production of medical devices and testing of medical equipment. Companies such as Siemens and Philips in India for the design, production, and testing of medical equipment, as a global delivery centre, and as a market for such products. According to the respondents, the entry of large multinationals into India in the medical devices segment as well as the emergence of world class corporate hospitals in India where such tests can be carried out are likely to drive these outsourcing possibilities. In this regard, the presence of bilateral investment treaties (BITS) between India and several EU member states (UK, France, Germany, Austria, Belgium, Denmark, Italy, Sweden, Poland, and Spain) could have a bearing on foreign direct investment and research and development related collaborations in the health care sector.

But collaboration was once again seen to be constrained by various factors. Linguistic differences and lack of mutual recognition constrain possibilities for staff exchange and deployment. Ethical regulations, liability and compensation-related concerns, and lack of international standards for registration of medical devices and technologies in India affect the scope for development and testing services for medical equipment and devices. There was also a general view that the EU has not been open to collaboration with India in the healthcare sector. Table 5 highlights constraints affecting specific areas where there are India-EU collaboration prospects.

**Table 5:** Constraints to collaboration in healthcare between India and the EU

Problem	Features and Implications
Political and social sensitivities	• Affect staffing and temporary movement of health personnel from India to EU countries
Recognition of qualifications	• Qualifications and experience of Indian health personnel not recognized in EU member countries • Re-certification and registration requirements impose additional costs on Indian doctors
Other regulatory issues	• Regulatory differences between India and the EU on ethics, liability, and production and testing

*Source*: Based on interviews

### Constraints in India

The primary research also revealed the presence of domestic constraints in India, which affect its exports of health services to the EU and also other developed country markets. These pertained to the lack of domestic regulatory frameworks or lack of enforcement of necessary regulations in India's health sector, particularly with regard to standards and accreditation of establishments and health personnel. Table 6 summarizes the main constraints within India that were highlighted by the interviews.

**Table 6:** Domestic Constraints to India's Health Services Exports to the EU

Constraint	Features and Implications
Accreditation and standards	• Absence of mutual recognition agreements with key markets, requiring Indian providers to undergo cumbersome certification and registration processes • Lack of recognition prevents Indian companies from drawing on overseas pool of medical manpower • Lack of standardization in medical and nursing training in India • No regulatory body in some areas (paramedics) • Authentication systems not perceived to be credible • Lack of international accreditation by most Indian healthcare establishments, preventing medical value travel, insurance portability, clinical trials outsourcing • Lack of registration, standardization and overseas recognition of alternative medicines and therapies • Lack of central laboratory accreditation that is recognized internationally (CAP)
Legal and regulatory framework	• Bureaucracy and delays in approval process for clinical trials • Delays in clearance for drug and sample shipments for testing • Multiple clearances required by CROs for undertaking clinical trials (from multiple Ministries) • Ethics approval process cumbersome as multiple committees involved • Absence of legislation in certain areas (movement of drugs within India, lack of procedural controls on use of medical devices) • Poor enforcement of registration for clinical trials • Slow regulatory clearances for bioequivalence studies • Lack of clarity in guidelines for biotechnology products • Jurisdictional issues about dispute resolution as lack of credible and efficient legal system in India • Gaps between India's clinical trials legislation and that of EU countries (e.g., requirement for pharmaceutical person for issuing drugs in the EU, not in India) • Concerns over violation of ethics by Indian CROs
Data protection	• Concerns over possible breach of data confidentiality after data submission to Indian regulatory body • Lack of strict firewalls for data leakage, guidelines on data exclusivity lacking, not strictly enforced
Insurance and litigation	• Lack of insurance portability, public or private from EU (related to lack of recognition of Indian qualifications and establishments) • Malpractice liability issues: concerns over dispute resolution, jurisdiction, appropriate compensation • Absence of insurance in India in emerging areas: clinical trials requiring insurance abroad at high cost
Other	• VAT and service tax charged on services of consultants monitoring clinical trials and reporting to client (export-oriented services usually exempt from service tax) • Delays in getting multiple entry visas for consultants monitoring clinical trials, short duration visas typical • Delays in bringing certain medical devices into India affecting medical device testing, research-related outsourcing

Source: Based on interviews

According to Indian and EU respondents, India needs to adopt a variety of regulatory measures and to align its own standards and regulations to international ones. This would enable India to leverage its capabilities in health services for exporting to and entering into collaborative arrangements with EU member countries as well as other developed regions.

## Conclusions

Certain broad policy directions emerge from the findings of this survey. Given the exclusion of health services from the EU's services directive, this is a challenging sector to discuss in any trade agreement with the EU. Moreover, given the sensitivities associated with commercialization of health care, and the likely difficulties in addressing issues such as recognition, data protection, or public attitudes in the EU in the short term, a cross-cutting approach based on cooperation might be more appropriate. Such collaboration would need to be on a selective basis, between selected institutions on both sides, between India and specific countries in the EU, and in selected opportunity segments.

India-EU cooperation in healthcare could involve institutional tie-ups, exchange of faculty, students, and trainees, research collaboration, cooperation on standards and recognition issues, and launching of joint programs and pilot projects between India and EU countries. Some specific areas for joint initiatives could include:

• Institutional tie-ups to facilitate telemedicine and medical value travel

• Partnerships and affiliations among labs and research centres to facilitate work in the area of clinical trials, and global recognition and certification of Indian labs

• Reciprocal health agreements with selected markets in the EU, along the lines of the agreements some of these countries have with non-member nations for treatments required during visits on emergency grounds

• Provisions to facilitate partnerships and collaboration among medical education and research institutions in India and the EU

• Pilot programs for staff deployment and exchange or medical value travel between select institutions on both sides, supported by collaborative programs in education, research, and training between selected prestigious hospitals, medical colleges, and centres in India and the EU

• Tie-ups between Indian hospitals/research centres and EU companies such as Siemens and Philips that develop medical devices and equipment, for commercial and academic reasons, also enabling Indian companies, research centres, and labs to partner in the engineering and design services work for the development of medical equipment

Over time, the demonstrated outcomes of such arrangements could provide the basis for expanding the bilateral relationship to include more providers and more EU member countries, and to cover more commercially-oriented opportunities. Collaboration could also help address longer term goals such as mutual recognition and changing public perceptions in the EU. For example, twinning programs, educational partnerships, and affiliations between institutions on both sides could help provide the basis for future discussions on equivalence of qualifications and mutual recognition. Likewise, tie-ups in the area of telemedicine could provide the basis for discussing the removal of outsourcing restrictions on patient data.

In parallel, the discussions would also need to focus on streamlining administrative procedures in the EU which act as impediments to Indian healthcare providers and to address specific issues such as data adequacy determination for India by EU data protection authorities. Attention would also be required on domestic measures concerning standards and data protection for India to effectively exploit its potential in health services exports, not only to the EU but more generally. It would also be important to link India's potential as an exporter of health services to its potential as a consumer of health care, such as for medical devices, drugs, diagnostic equipment, and as a market for foreign investment by EU companies and hence the win-win possibilities. Greater awareness would also need to be created on both sides about the competencies in each other's markets.

## Competing interests

The author declares that they have no competing interests.

## Authors' contributions

The author carried out the entire study. She conceived and designed the interviews and their customization for different respondents. She did the entire data collection, analysis, and interpretation of findings. She drafted the entire manuscript and subsequent revisions.
